# Quantification of maize brace root formation after vertical stalk displacement

**DOI:** 10.17912/micropub.biology.001189

**Published:** 2024-04-02

**Authors:** Jonathan Reneau, Noah Ouslander, Erin E Sparks

**Affiliations:** 1 Department of Plant and Soil Sciences, University of Delaware, Newark, Delaware, United States

## Abstract

Maize brace roots develop from aboveground stem nodes in both upright and vertically displaced stalks. The cues that trigger brace root development after displacement are unknown. Possibilities include disturbance of the belowground roots, gravity, moisture, physical interaction, or node anatomical changes. We show that brace root formation occurs at all growth stages, with more nodes producing brace roots when plants are displaced at later growth stages. This occurs with the underground roots intact, without moisture accumulation and without physical interaction. We propose that the formation of brace roots after vertical stalk displacement is most likely due to gravity or anatomical changes at the node.

**Figure 1. Brace root development after vertical stalk displacement:**
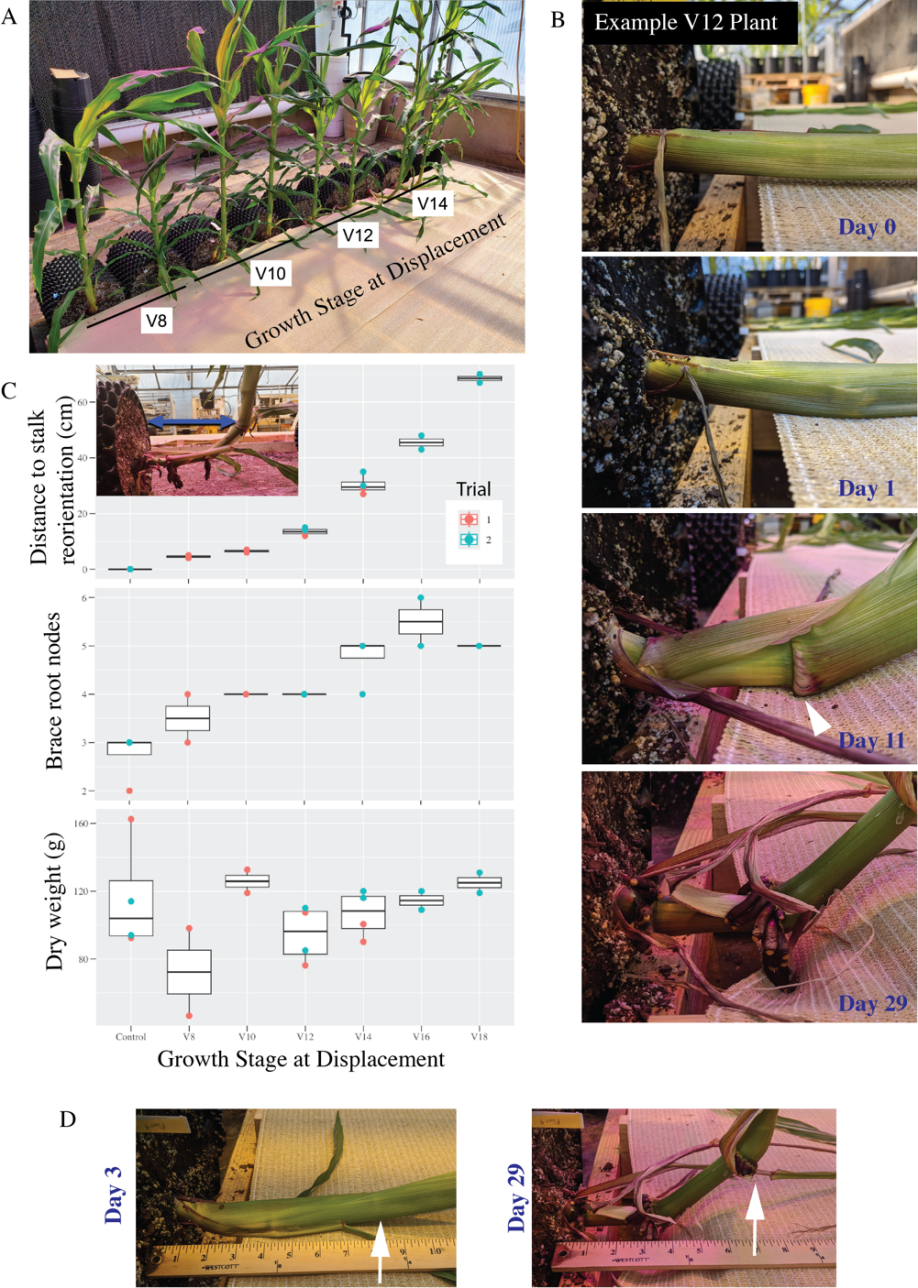
A) Trial 1 plants shown after the recovery period. B) An example V12 plant over selected time after displacement. At Day 0, the stalk is horizontal and not touching the underlying shade cloth. At Day 1, the stalk is already bending at the node. By Day 11, the first split in the leaf sheath reveals a brace root emerging (white arrowhead). By Day 29, brace roots have completely emerged from the node and are pushing against the shade cloth. C) At progressively older growth stages, there is a larger Euclidean distance to reorientation (the distance between the soil and the vertical section stalk, inset image blue arrow). Concurrent with the larger distances, there are more nodes that produce brace roots after displacement. There was no notable difference in dry weight among the different stages of displacement. D) A node that was not in contact with the shade cloth (Day 3, white arrow) still produced brace roots (Day 29, white arrow).

## Description

With a changing global climate, agricultural production is threatened by increasingly severe storm systems. These storms can induce crop mechanical failure called lodging (Rajkumara, 2008) . Lodging refers to an event that displaces the plant from its vertical position. This displacement can be due to stalk buckling or breaking (i.e., stalk lodging or greensnap) or due to root failure (i.e., root lodging) (Erndwein et al, 2020) . In maize ( *Zea mays* ), root lodging has significant impacts on production (Tirado et al., 2021; Hostetler et al., 2022) . However, maize also has specialized aboveground roots called brace roots that support the plant and limit root lodging (Reneau et al., 2020; Hostetler et al., 2022; Obayes et al., 2022) .

Understandably, most studies have focused on the plant phenotypes and characteristics that prevent root lodging (e.g., Xue et al., 2020; Zhang et al., 2021; Hostetler et al., 2022; Zheng et al., 2023). However, plant recovery after root lodging is also quite interesting. Root lodging generally does not kill the plant and plants have the remarkable ability to reorient their stalks to vertical, a process called “goose-necking”. The main limitation to harvesting root lodged maize is that a mechanical combine harvester cannot pick up vertically displaced stalks. One study of manual root lodging at different growth stages showed progressive reductions in yield that coincided with increased “goose-necking” at later growth stages (Carter & Hudelson, 1988). What is not reported in this study is the development of brace roots from the stem nodes after stalks have been displaced from vertical (Sparks, 2023) .

The development of roots from displaced stalks is not unique to maize, but is a process that has been poorly studied in general. Specifically, it is unknown how displacement triggers root development. We propose several hypotheses for how this happens: 1) The disruption or damage of the underground root system triggers stalk-borne root development. 2) The shift in the perception of gravity triggers stalk-borne root development through gravitropic responses. 3) The proximity of the stalk to moisture triggers stalk-borne root development through hydrotropic responses. 4) The physical interaction with the soil (assuming the stalk touches the ground) triggers stalk-borne root development through thigmotropic responses. 5) The changing nodal anatomy that occurs during the stalk reorientation triggers stalk-borne root development. In *Brachypodium distachyon* the development of stalk-borne roots has been attributed to the physical interaction with the soil triggering a thigmotropic response (Nam et al., 2020) . However, we previously posited that this is likely not the case for maize since we observe brace roots forming when the stalk is not in contact with the ground (Sparks, 2023) .

In this study, we attempt to narrow the possible hypotheses about what triggers brace root growth after vertical displacement. Specifically, we built a platform to displace maize stalks from vertical at different growth stages ( Figure 1A ). To eliminate the possibility of damage to the underground root system and test hypothesis 1 above, whole pots were rotated 90° from the vertical position, leaving the underground root system intact. Further, to limit moisture accumulation and test hypothesis 3 above, a breathable shade cloth was installed to form the surface of the platform. Lastly, stalks were placed close to the platform, but due to variation in the centeredness of the stalk within the pot the lowest portion of the stalks had a variable interaction with the platform. This then created a variable physical interaction environment with some stalks in direct contact with the platform and other stalks hovering above the platform. Thus, our system is considering only the signals from gravity and changes in nodal anatomy due to the vertical reorientation of stalks.

Assessment of the plants at the VT-stage showed “goose-necking” and increased brace root node number in all plants regardless of the growth stage at displacement. Using a plant that was displaced at the V12 stage as an example, stalks start to reorient the growth pattern to vertical as soon as Day 1 post-displacement ( Figure 1B ). At Day 11, the first brace root appeared to break through the overlying leaf sheath ( Figure 1B ). By Day 29, brace roots are fully emerged and pushing against the shade cloth ( Figure 1B ).

The degree of vertical stalk reorientation was determined by measuring the Euclidean distance between the soil and vertical section of stalk (see inset Figure 1C ). Consistent with previous reports, the distance to stalk reorientation increased with the displacement of plants at later growth stages ( Figure 1C ). Additionally, the number of nodes producing brace roots was greater in displaced plants compared to the upright control plants for all growth stages ( Figure 1C ). The number of nodes producing brace roots increased with the displacement of plants at later growth stages, but appeared to plateau when the plants were displaced at the V16-V18 stage. Despite these developmental changes, there was no notable impact on the dry mass of the plants ( Figure 1C ). However, the sample size is limited, which limits the interpretation of any small differences that may be present.

These results show that brace root development after displacement occurs within an intact belowground root system and in the absence of moisture. Further, the observation of stem nodes overtime showed that nodes that never contacted the shade cloth still had the development of brace roots from the nodes ( Figure 1D ). Thus, the development of brace roots after displacement is unlikely due to physical interaction.

Collectively, these results show that brace root development from stem nodes after displacement occurs at all growth stages analyzed. Considering the original five hypotheses, this data suggests that the signals that trigger brace root development upon displacement are either triggered by gravity or changes in the nodal anatomy during stalk reorientation. These signals remain to be fully investigated, but raise additional questions such as – is gravity signaling within stalks or roots responsible for triggering brace root development? Is there a physiological or structural advantage for a plant to form brace roots after displacement? Speaking outside the context of agriculture, this may be an ancient survival and propagation strategy that is retained in modern maize.

## Methods

A platform to facilitate this study was built with a wood frame (3.6 m long x 2.4 m wide x 0.14 m tall) and covered with shade cloth (Windscreen4Less; 75% Wind Screen Sun Block Fabric). The shade was secured with wooden paint stir sticks and screws on the 2.4 m side of the frame. B73 maize seeds were planted in 21.1 L propagation air-pots (Air-Pot Superoots THAP7) with PRO-MIX BK55 soil. Air-pots were chosen to enable watering in the displaced position and to promote maize growth to later stages. Plants were grown in the greenhouse under 16 h daylight conditions. Natural daylight was supplemented with overhead high pressure sodium lamps to 50,000 lux (approximately 925.93 µmol/m ^-2^ /s ^-1^ PPFD (photosynthetic photon flux density)). Temperatures were set at 26.7 °C during the day and 21.1 °C at night. Plants were watered with Peters Excel 21-5-20 multipurpose fertilizer when the soil in the upright plants dried to ~3 cm.

Two separate trials were conducted. In the first trial, two plants each were displaced at the V8, V10, V12, and V14 stage and analyzed at the VT stage. In the second trial, two plants each were displaced at the V12, V14, V16, and V18 stage and analyzed at the VT stage. For each trial two plants were maintained in the upright position as controls and also analyzed at VT.

Images were taken of each plant and the length to reorientation (distance between the vertical length of stem and the soil surface) and number of nodes producing brace roots was extracted from images. Aboveground plant material was dried, and dry weight was recorded. Data were analyzed with R Studio (version 2023.12.1+402) and R (version 4.3.2) and graphed with ggplot2 (version 3.5.0).
